# The characterization of Parkinson's disease related pain based on the classification system: The severity of neuropathic pain and the clinical profile of nociplastic pain

**DOI:** 10.1016/j.prdoa.2026.100490

**Published:** 2026-07-27

**Authors:** Toshiki Tezuka, Tomonori Nukariya, Shohei Okusa, Ryo Ueda, Hiroki Saegusa, Ryotaro Okochi, Yuto Sakai, Yoshihiro Nihei, Jin Nakahara, Morinobu Seki

**Affiliations:** a Department of Neurology, Keio University School of Medicine, Tokyo, Japan; b Parkinson's Disease Center, Keio University Hospital, Tokyo, Japan; c Center for Parkinson's disease Research, Keio University, Tokyo, Japan; d Office of Radiation Technology, Keio University Hospital, Tokyo, Japan; e Department of Rehabilitation Medicine, Keio University School of Medicine, Tokyo, Japan

**Keywords:** Parkinson's disease, Pain classification system, Neuropathic pain, Nociplastic pain, Free-water imaging, Neurite orientation dispersion and density imaging

## Abstract

**Introduction:**

Pain significantly impairs quality of life in Parkinson's disease (PD). While the PD Pain Classification System (PD-PCS) categorizes PD-related pain into nociceptive, neuropathic, and nociplastic pain, the clinical and structural correlates of these subtypes, particularly nociplastic pain, remain poorly characterized. The objective of this study was to elucidate the distinct features of each PD-related pain subtype.

**Methods:**

This single-center cross-sectional study involved a two-stage PD-PCS-based evaluation. First, 192 PD patients were assessed for the prevalence of pain subtypes and their associations with demographic and clinical variables. Second, 40 PD patients with pain underwent detailed physician-led assessments using validated motor and non-motor scales, including MDS-UPDRS, KPPS, KPPQ, NMSS, HADS, PDSS-2, PDQ-8, WOQ-9, and VAS. Structural and diffusion MRI analyses, including free-water imaging and neurite orientation dispersion and density imaging, were performed in a subset of participants to explore pain-related neural correlates.

**Results:**

Among 192 PD patients in the first survey, 107 (55.7%) reported pain, classified as nociceptive (37.4%), neuropathic (18.7%), nociplastic (23.4%), or PD-unrelated pain (20.6%). Compared with nociplastic pain, neuropathic pain showed higher PD-PCS scores (39.8 ± 29.2 vs 14.3 ± 16.9; *p* = 0.0014) and higher King's Parkinson's Pain Questionnaire scores (5.8 ± 2.7 vs 2.9 ± 1.5; *p* = 0.00014). Compared with nociceptive pain, neuropathic pain also showed higher questionnaire scores (5.8 ± 2.7 vs 3.8 ± 2.1; *p* = 0.013). In the second survey, neuropathic pain was associated with greater motor complications than nociplastic pain (MDS-UPDRS Part IV: 10.2 ± 2.9 vs 3.9 ± 3.4; *p* = 0.029). In the supplementary MRI analysis, neuropathic pain showed distinct microstructural abnormalities in pain-processing neural networks. Conversely, nociplastic pain showed no distinctive motor/non-motor features or detectable MRI abnormalities, despite a comparable negative impact on quality of life.

**Conclusion:**

Neuropathic pain in PD represents a severe phenotype linked to both motor fluctuations and structural brain changes, underscoring the need for mechanism-specific management strategies. By contrast, the absence of defining clinical or structural markers in nociplastic pain highlights it diagnostic challenge and suggests that current assessment tools may insufficiently capture this pain mechanism. These findings emphasize the need for multidimensional and mechanism-oriented approaches to PD-related pain in clinical practice.

## Highlights

• Characterizing Parkinson's disease related pain by Pain Classification System.
• Neuropathic pain exhibited more severe features and structural brain MRI changes.
• Nociplastic pain showed no distinct motor or non-motor features and no MRI changes.
• This first characterization of nociplastic pain highlighted its diagnostic complexity.

## 1 Introduction

Approximately 40%–85% of individuals with Parkinson's disease (PD) experience pain that negatively affects their quality of life [1], [2]. Despite the prevalence and burden on daily living, pain in PD is often under-reported and inadequately assessed due to the lack of validated PD-specific pain scales, leading to suboptimal management [3]. The Parkinson's Disease Pain Classification System (PD-PCS), developed in 2021 by pain specialists and movement-disorder experts to address mechanism-based pain diagnoses and management, was validated in an international multicenter study with a retest step [4]. The PD-PCS distinguishes PD-related pain from PD-unrelated pain based on the pain's association with PD manifestations, and it incorporates clinical and pathophysiological descriptors (neuropathic, nociceptive, and nociplastic pain) in agreement with the International Association for the Study of Pain classification [5].

Nociceptive pain results from an activation of nociceptors by mechanical or thermal stimuli related to an actual or potential tissue injury. Neuropathic pain arises from a lesion or disease of the peripheral or central somatosensory systems, with specific qualities (tingling, burning, or electric-shock-like) and a neurologically plausible distribution. Nociplastic pain comprises conditions in which the nociceptive system is overactive without a somatosensory lesion or peripheral nociceptor activation. Applying these general pain subtype definitions in the context of PD can be challenging, as pain in patients with PD often intertwines with diverse motor and non-motor symptoms. PD-related nociplastic pain is thought to occur in the context of the non-motor off state, restless legs syndrome (RLS), dopamine agonist withdrawal syndrome (DAWS), dopamine dysregulation syndrome (DDS), and other neuropsychiatric manifestations [4]. However, given the heterogeneity of nociplastic pain and incomplete understanding of its neuroanatomical basis in PD, its characterization remains to be clarified [6], [7].

The application of the mechanism-based PD-PCS together with supplementary MRI assessments was hypothesized to reveal distinct clinical and structural characteristics of PD-related pain subtypes, particularly aiding characterization of nociplastic pain. To test this hypothesis, PD patients underwent PD-PCS-based evaluations integrating multiple motor and non-motor indicators with supplementary MRI assessments.

## 2 Patients and methods

### 2.1 Design and patients

This single-center cross-sectional observational study was conducted in Japan. According to the study objectives, two PD-PCS-based surveys were conducted in PD patients treated at Keio University Hospital during different periods: January 2022–December 2024 and February–April 2025. The first survey broadly assessed the prevalence of each pain type and its associations with clinical features derived from patient questionnaires and medical records. To further characterize each pain type, the second survey comprehensively evaluated motor and non-motor symptoms through physician interviews. Because the two surveys were conducted at the same institution, some eligible patients participated in both surveys.

PD patients aged ≥20 years who met the Movement Disorder Society (MDS) diagnostic criteria for clinically established or probable PD and were treated at our hospital were included [8]. These criteria were selected to ensure a clinically well-defined PD cohort suitable for pain phenotyping. PD patients were included in the first survey regardless of the pain status to investigate pain frequency. Patients with a clinically established cause of pain unrelated to PD, such as orthopedic disorders, were excluded from the second survey. Common exclusion criteria were: [1] other neurological disorders or undiagnosed/drug-induced parkinsonism, to avoid diagnostic confounding; [2] refusal to participate; and [3] severe dementia precluding reliable questionnaire completion. All evaluations were performed during the “on” state to reduce the influence of motor fluctuations on assessments.

The Ethics Committee for Human Research of the Keio University School of Medicine approved the study design and protocol (#N20231166). Written informed consent was waived in accordance with national ethical guidelines. An opt-out procedure disclosed the study information on the institutional website to allow non-participation.

### 2.2 Assessments

#### 2.2.1 Motor and non-motor symptom assessments

The patient's age, sex, and disease duration data were obtained from their medical records. Each patient's levodopa equivalent daily dose (LEDD) was calculated using their medications [9]. Motor severity was assessed using the Hoehn and Yahr (HY) stage (stages 1–5; higher stages indicate more advanced disease) and the Movement Disorder Society Unified Parkinson's Disease Rating Scale (MDS-UPDRS). The MDS-UPDRS evaluates non-motor experiences of daily living (Part I, 0–52), motor experiences of daily living (Part II, 0–52), motor examination (Part III, 0–132), and motor complications including dyskinesia and wearing-off (Part IV, 0–24); higher scores indicate greater severity. Non-motor burden was assessed with the Non-Motor Symptoms Scale (NMSS; 0–360, higher scores indicate greater burden) [10]. Cognition was evaluated using the Mini-Mental State Examination (MMSE; 0–30, lower scores indicate worse cognition) [11] and the Montreal Cognitive Assessment, Japanese version (MoCA-J; 0–30, lower scores indicate worse cognition) [12]. Depression and anxiety were assessed using the Hospital Anxiety and Depression Scale (HADS; 0–42 total, higher scores indicate greater symptoms) [13]. Sleep disturbance was evaluated using the Parkinson's Disease Sleep Scale-2 (PDSS-2; 0–60, higher scores indicate worse sleep disturbance) [14] and the REM Sleep Behavior Disorder (RBD) Screening Questionnaire, Japanese version (RBDSQ-J; 0–13, higher scores indicate greater likelihood of RBD) [15]. Olfactory function was assessed using the Odor Stick Identification Test for Japanese (OSIT-J; 0–12, lower scores indicate worse olfactory function) [16]. Quality of life was assessed using the Parkinson's Disease Questionnaire-8 (PDQ-8; 0–32, higher scores indicate worse quality of life) [17], and wearing-off phenomena were evaluated using the nine-item Wearing-off Questionnaire (WOQ-9; 0–9, higher scores indicate greater wearing-off burden) [18].

#### 2.2.2 Pain assessments

Pain was evaluated using the PD-PCS [4], King's Parkinson's Pain Scale (KPPS; higher scores indicate greater pain severity) [19], [20], King's Parkinson's Pain Questionnaire (KPPQ; higher scores indicate broader pain burden) [20], [21], and a visual analog scale (VAS; 0–100, higher scores indicate greater pain intensity). The web-based PD-PCS was translated into Japanese by the author involved in the Japanese translation of the KPPS and KPPQ [20] and administered with physicians' supplementary explanations.

In the first survey, the PD-PCS, the KPPQ, and the MDS-UPDRS Part I (Pain and other sensations) were administered. The patients whose scores were zero on all three assessments were classified as the No-pain group. Some of the patients also completed the MMSE, MoCA-J, OSIT-J, RBDSQ-J, and ^123^I-metaiodobenzylguanidine (MIBG) myocardial scintigraphy. In the second survey, the PD-PCS, KPPS, KPPQ, MDS-UPDRS Parts I–IV, NMSS, HADS, PDSS-2, PDQ-8, WOQ-9, and VAS were administered.

### 2.3 Image acquisition

To complement the survey findings, brain MRI was performed in a subset of patients within 1 year of participation. The diffusion-weighted imaging (DWI) processing pipeline was used as described [22]. Brain MRI was performed on a Discovery MR750 3.0-T system (GE Healthcare, Milwaukee, WI, USA) with a 32-chanel head coil. Structural data were acquired using a three-dimensional T1-weighted BRAVO sequence (repetition time [TR] 6.8 ms, echo time [TE] 3.0 ms, flip angle [FA] 8°) with a 256 × 256 matrix and a 230 × 230-mm field of view (FOV). The reconstruction voxel size was 0.90 × 0.90 × 1.00 mm^3^, and 200 sagittal sections (1-mm slice thickness) were obtained. Multi-shell DWI data were collected with a spin-echo single-shot echo-planer imaging (EPI) sequence (TR/TE/FA: 6500 msec/75 msec/90°), with an asset parallel-imaging scheme (reduction factor of 2.0). The diffusion-weighted images were acquired with b-values of 0, 1000, and 3000 s/mm^2^.

Thirty diffusion-encoding directions were acquired at b = 1000 s/mm^2^ and 60 directions were acquired at b = 3000 s/mm^2^, all axially oriented. For susceptibility-distortion correction, two b = 0 s/mm^2^ images were additionally acquired with reversed phase-encoding directions (posterior–anterior and anterior–posterior), whereas all DWI volumes were collected using a single fixed phase-encoding direction. Images were acquired with a 128 × 128 matrix and a 320 × 320-mm FOV. The reconstructed voxel size was 2.5 × 2.5 × 2.5 mm^3^. Fifty-six axial sections (2.5-mm slice thickness) were obtained. All images were visually checked for scanner artifacts and anatomical anomalies.

### 2.4 FreeSurfer data analysis

The original DICOM data were converted to NIfTI format with the use of an MRIcron image viewer; non-brain structures were removed by using the FMRIB software library (FSL; http://www.fmrib.ox.ac.uk/fsl). FreeSurfer ver. 7.4.0 was used for the automated volumetric and surface-based segmentation of T1-weighted images with the recon-all pipeline, including automated segmentation (aseg) and cortical parcellation (aparc).

For the extraction of pain-related brain regions in detail, probabilistic atlases available in FreeSurfer were applied: amygdala nuclei, brainstem substructures, hippocampal subfields, and thalamic nuclei. No manual modifications were required because all segmentations were visually confirmed. Regional measures of volume, surface area, and cortical thickness values were extracted from each segmented region. To account for inter-individual differences in head size, all regional volumes and surface areas were normalized by the estimated total intracranial volume.

### 2.5 DWI processing and free-water imaging (FWI) analysis

MRtrix3 (https://www.mrtrix.org/) was used for DWI preprocessing, including denoising and removal of the Gibbs artifact. In the distortion-corrected pipeline, susceptibility-induced EPI distortions were corrected with the FSL TOPUP tool, using two reverse phase-encoded b = 0 s/mm^2^ images (posterior–anterior and anterior–posterior) to estimate the off-resonance field. The resulting field was then supplied to FSL EDDY to correct eddy current-related and motion-related artifacts for all diffusion-weighted volumes. Subsequently, FSL FMRIB's Diffusion Toolbox diffusion function was used to fit the diffusion tensor and estimate the related indices of white-matter integrity. The diffusion MRI time series were co-registered to the T1-weighted images.

DIPY (https://nipy.org/packages/dipy/index.html) was used to analyze the preprocessed data. In each voxel, the signal was fitted to a two-compartment model, including a free-water compartment (isotropic tensor) and a tissue compartment (free-water-corrected tensor). The estimated parameters were the fractional volume of the free-water compartment (i.e., the free-water measure) and the tensor of the tissue compartment. The free-water measure expresses the relative free-water contribution in each voxel (range: 0–1), and the tissue tensor reflects the microstructure after the removal of the free-water signal.

Tissue-compartment measures (denoted by the subscript ‘t’), including the mean diffusivity (md), fractional anisotropy (fa), radial diffusivity (rd), and axial diffusivity (ad) were calculated based on the tensor decomposition of the free-water-corrected tensor. The neurite orientation dispersion and density imaging (NODDI) metrics (fitted intracellular volume fraction [ficvf] and isotropic volume fraction [fiso]) were derived from the preprocessed diffusion MRI volumes using the Matlab NODDI toolbox (ver. 1.0.5, Matlab R2019b) for computation and fitting of the NODDI microstructure model. The b0 and 3D-T1-weighted images, along with the resultant diffusion images, were co-registered using advanced normalization tools and normalized to the Montreal Neurological Institute 152 template space.

### 2.6 Statistical analyses

Between-group comparisons of clinical and MRI variables were conducted using Welch's *t*-test or Mann-Whitney *U* test for two groups, a one-way analysis of variance or the Kruskal-Wallis test for three groups, and the chi-square or Fisher's exact test, as appropriate. For three-group comparisons, Tukey's or Dunn's test was used for the post hoc analysis. Associations with PD-PCS scores were examined using Pearson's or Spearman's correlation, depending on data normality. To account for covariates in the MRI analysis, an analysis of covariance (ANCOVA) or rank-based ANCOVA was applied, depending on normality. Sex and age were covariates in all of the MRI analyses, and MDS-UPDRS Part IV was included in comparisons of MRI metrics between the patients with and without pain. R ver. 4.5.1 (R Development Core Team, 2025) was used for the statistical analyses. Significance was set at *p* < 0.05 after Bonferroni correction for multiple comparisons.

## 3 Results

### 3.1 Characteristics of the patients in the first survey (supplementary Table.1)

As summarized in Supplementary Table.1, 199 PD patients were examined in the first survey; seven patients were excluded due to incomplete questionnaires, leaving 192 patients' cases for analysis. These patients were 112 males (58.3%) and 80 females (41.7%); mean ± standard deviation values: age 72.7 ± 8.9 years, age at onset 64.5 ± 10.1 years, disease duration 8.2 ± 5.8 years, HY stage 2.8 ± 0.9, and LEDD 690 ± 439 mg. The MMSE, MoCA-J, OSIT-J, RBDSQ-J, and MIBG scintigraphy were performed in a subset of these patients.

### 3.2 Evaluation of PD-related pain based on the PD-PCS and other scales

#### 3.2.1 Frequency of each pain type (Table.1)

Based on the patients' results on MDS-UPDRS Part I-9 (pain and other sensations), KPPQ, and PD-PCS, the 192 PD patients were divided into No-pain group (*n* = 85, 44.3%) and Pain group (*n* = 107, 55.7%). According to PD-PCS, the Pain group was further divided into PD-related pain group (n = 85, 79.4%) and PD-unrelated pain group (*n* = 22, 20.6%). In addition, the PD-related pain group (n = 85) was categorized into three subtypes: Nociceptive pain (*n* = 40, 37.4%), Neuropathic pain (*n* = 20, 18.7%), and Nociplastic pain (*n* = 25, 23.4%).

**Table 1:** Comparison of clinical data between the ‘No pain’ and ‘PD-related pain’ group, and among the ‘Nociceptive pain’, ‘Neuropathic pain’, and ‘Nociplastic pain’ group in the first survey.

	No pain	PD-related pain		PD-related Pain					PD-unrelated pain
		values	*p*-value (vs No pain)	Nociceptive pain	Neuropathic pain	Nociplastic pain	*p*-value (in PD-related pain)	Corrected *p*-value (post-hoc)	
Number (Ratio to total number *N* = 192, %)	85 (44.3%)	85 (44.3%)	–	40 (20.8%)	20 (10.4%)	25 (13.0%)	–	–	22 (11.5%)
Proportion among PD patients with pain (N = 107)	–	79.4%	–	37.4%	18.7%	23.4%	–	–	20.6%
Sex (Male: Female)	53: 32	48: 37	0.43	22: 18	10: 10	16: 9	0.62	–	11: 11
Age	73.9 ± 8.5	71.4 ± 9.6	0.077	70.5 ± 10.4	71.8 ± 8.2	72.4 ± 9.4	0.72	–	73.0 ± 7.2
Age at onset	66.6 ± 9.5#	62.2 ± 10.6#	0.0047	61.7 ± 12.0	60.9 ± 7.6	64.0 ± 10.3	0.34	–	65.1 ± 8.7
Disease duration	7.3 ± 5.7*	9.2 ± 5.8*	0.012	8.8 ± 5.2	10.9 ± 7.2	8.4 ± 5.2	0.38	–	7.9 ± 6.1
HY stage	2.7 ± 1.0#	3.0 ± 0.9#	0.007	3.0 ± 0.9	3.4 ± 0.9	2.9 ± 0.8	0.10	–	2.7 ± 0.9
LEDD (mg)	567 ± 429$	823 ± 427$	< 0.001	832 ± 394	938 ± 522	716 ± 385	0.31	–	653 ± 394
PD-PCS score	0$	24.4 ± 25.1$	< 0.001	23.0 ± 24.0	39.8 ± 29.2#	14.3 ± 16.9#	0.0022	0.0014 (Neuropathic vs Nociplastic)	NA
KPPQ total score	0$	4.0 ± 2.3$	< 0.001	3.8 ± 2.1*	5.8 ± 2.7*$	2.9 ± 1.5$	0.00022	0.00014, 0.013 (Neuropathic vs Nociplastic, Nociceptive)	1.8 ± 1.1
1. Pain around joints	0	69 (81.1%)	–	31 (77.5%)	19 (95.0%)	19 (76.0%)	0.20	–	17 (77.3%)
2. Pain related to a specific internal pain	0	20 (23.5%)	–	10 (25.0%)	8 (40.0%)*	2 (8.0%)*	0.031	0.043 (Neuropathic vs Nociplastic)	3 (13.6%)
3. Generalised non-specific pain in stomach area	0	18 (21.1%)	–	8 (20.0%)	7 (35.0%)	3 (12.0%)	0.19	–	2 (9.1%)
4. Non-specific pain deep within the body	0	24 (28.2%)	–	12 (30.0%)	8 (40.0%)	4 (16.0%)	0.20	–	1 (4.5%)
5. Pain related to abnormal involuntary movements	0	17 (20.0%)	–	5 (12.5%)	6 (30.0%)	6 (24.0%)	0.23	–	0
6. Painful muscle cramps in a specific region during “off” periods	0	38 (44.7%)	–	24 (60.0%)*	11 (55.0%)	3 (12.0%)*	0.015	0.017 (Nociceptive vs Nociplastic)	0
7. Generalised pain during “off” periods	0	26 (30.6%)	–	11 (27.5%)	8 (40.0%)	7 (28.0%)	0.098	–	0
8. PLM or RLS-associated pain	0	39 (45.9%)	–	20 (50.0%)	12 (60.0%)	7 (28.0%)	0.078	–	6 (27.2%)
9. Pain related to difficulties when turning in bed at night	0	30 (35.3%)	–	12 (30.0%)	11 (55.0%)	7 (28.0%)	0.11	–	3 (13.6%)
10. Pain when chewing	0	8 (9.4%)	–	4 (10.0%)	2 (10.0%)	2 (8.0%)	1.00	–	1 (4.5%)
11. Pain related to grinding teeth during the night	0	3 (3.5%)	–	2 (5.0%)	0	1 (4.0%)	0.80	–	1 (4.5%)
12. Burning sensation in a mouth	0	6 (7.1%)	–	3 (7.5%)	2 (10.0%)	1 (4.0%)	0.86	–	0
13. Burning pain in the limbs	0	13 (15.3%)	–	2 (5.0%)*	7 (35.0%)*	4 (16.0%)	0.01	0.013 (Nociceptive vs Neuropathic)	0
14. Shooting pain/pins and needles down the limbs	0	30 (35.3%)	–	9 (22.5%)#	14 (70.0%)#*	7 (28.0%)*	0.00091	0.0018, 0.022 (Neuropathic vs Nociceptive, Nociplastic)	5 (22.7%)
MDS-UPDRS Part I; Pain and other sensations	0$	2.1 ± 0.9$	< 0.001	2.1 ± 0.9	2.5 ± 0.9	2.0 ± 0.8	0.22	–	1.6 ± 0.8
MMSE	27.1 ± 3.3 (N = 38)	27.3 ± 2.6 (N = 37)	0.94	27.2 ± 2.8 (N = 16)	27.8 ± 3.2 (N = 9)	27.0 ± 1.8 (N = 12)	0.46	–	23.2 ± 5.7 (N = 4)
MoCA-J	23.1 ± 3.8 (*N* = 38)	23.5 ± 3.7 (*N* = 37)	0.83	24.4 ± 4.4 (*N* = 16)	23.7 ± 4.0 (*N* = 9)	22.2 ± 2.1 (*N* = 12)	0.31	–	19.0 ± 7.3 (*N* = 4)
OSIT-J	3.8 ± 3.0 (*N* = 70)	4.0 ± 2.6 (*N* = 63)	0.46	3.7 ± 2.8 (*N* = 31)	4.6 ± 2.7 (*N* = 14)	4.0 ± 2.1 (N = 18)	0.36	–	5.2 ± 3.7 (N = 18)
RBDSQ-J	3.7 ± 2.5$ (*N* = 76)	5.8 ± 3.4$ (*N* = 75)	< 0.001	5.8 ± 3.4 (*N* = 33)	6.1 ± 3.1 (*N* = 18)	5.6 ± 3.5 (N = 24)	0.80	–	4.8 ± 2.5 (*N* = 21)
MIBG scintigraphy; positive, negative	pos: 60, neg: 13	pos: 63, neg: 11	–	pos: 31, neg: 4	pos: 16, neg: 1	pos: 16, neg: 6	–	–	pos: 10, neg: 5

Comparison of clinical data between the ‘No pain’ and ‘PD-related pain’ group, and among the ‘Nociceptive pain’, ‘Neuropathic pain’, and ‘Nociplastic pain’ group in the first survey. All data are presented as mean ± standard deviation or n (%). The signs of *, #, and $ means that *p*-value is <0.05, < 0.01, and < 0.001, respectively.

HY, Hoehn and Yahr; KPPQ, King's Parkinson's Pain Questionnaire; LEDD, levodopa equivalent daily dose; MDS-UPDRS, Movement Disorder Society Unified Parkinson's Disease Rating Scale; MIBG, metaiodobenzylguanidine; MMSE, Mini-Mental State Examination; MoCA-J, Japanese version of the Montreal Cognitive Assessment; NA, not available; OSIT-J, Odor Stick Identification Test for Japanese; PD, Parkinson's disease; PD-PCS, PD Pain Classification System; PLM, periodic limb movement; RBDSQ-J, Japanese version of the REM Sleep Behavior Disorder Screening Questionnaire; RLS, restless legs syndrome.

#### 3.2.2 Comparisons of the no-pain and PD-related pain groups (Table.1)

Compared to the No-pain group, the PD-related pain group had a significantly younger onset age (62.2 ± 10.6 vs 66.6 ± 9.5 years, *p* = 0.0047), significantly longer disease duration (9.2 ± 5.8 vs 7.3 ± 5.7 years, *p* = 0.012), significantly more advanced HY stage (3.0 ± 0.9 vs 2.7 ± 1.0, *p* = 0.007), significantly higher LEDD (823 ± 427 vs 567 ± 429 mg/day, *p* < 0.001), and significantly higher RBDSQ-J scores (5.8 ± 3.4 vs 3.7 ± 2.5, *p* < 0.001). These findings suggest that PD-related pain was associated with greater disease burden, more complex dopaminergic treatment requirements, and increased REM sleep behavior disorder symptoms. No significant between-group differences were observed for sex (*p* = 0.43), age (*p* = 0.077), or MMSE (*p* = 0.94), MoCA-J (*p* = 0.83), and OSIT-J scores (*p* = 0.46), indicating no clear differences in global cognition or olfactory function.

#### 3.2.3 Comparisons among PD-related pain subtypes (Table.1)

Patients in the Neuropathic pain group showed higher PD-PCS scores than those in the Nociplastic pain group (39.8 ± 29.2 vs 14.3 ± 16.9, *p* = 0.0014) and higher KPPQ total scores than both the Nociplastic pain group (5.8 ± 2.7 vs 2.9 ± 1.5, *p* = 0.00014) and the Nociceptive pain group (5.8 ± 2.7 vs 3.8 ± 2.1, *p* = 0.013). These findings indicate a greater overall pain burden in the Neuropathic pain group. Symptom-level analysis of the KPPQ further demonstrated that the Neuropathic pain group more frequently reported ‘Pain related to a specific internal pain’ (40.0% vs 8.0%, *p* = 0.043 vs Nociplastic pain), ‘Burning pain in the limbs’ (35.0% vs 5.0%, *p* = 0.013 vs Nociceptive pain), and Shooting pain/pins and needles down the limbs' (70.0% vs 22.5%, *p* = 0.0018 vs Nociceptive pain; vs 28.0%, *p* = 0.022 vs Nociplastic pain), consistent with a clinically typical neuropathic pain phenotype. In contrast, the Nociceptive pain group more frequently reported painful muscle cramps in a specific region during “off” periods than the Nociplastic pain group (60.0% vs 12.0%, *p* = 0.017), suggesting a stronger association with motor fluctuation-related musculoskeletal pain. No significant differences among the three subgroups were observed in sex (*p* = 0.62), age (*p* = 0.72), age at onset (*p* = 0.34), disease duration (*p* = 0.38), HY stage (*p* = 0.10), LEDD (*p* = 0.31), MMSE (*p* = 0.46), MoCA-J (*p* = 0.31), OSIT-J (*p* = 0.36), or RBDSQ-J (*p* = 0.80), indicating that pain subtype differences were not explained by general demographic or disease-severity factors.

#### 3.2.4 Comparisons of the PD-related pain subtypes according to the presence or absence of clinical features (supplementary Table.2)

Patients were stratified according to (i) MIBG scintigraphy findings (positive: heart-to-mediastinum uptake ratio < 2.2; negative: ≥2.2), (ii) probable REM sleep behavior disorder (RBDSQ-J ≥ 6), (iii) body-first versus brain-first clinical subtype based on the temporal relationship between RBD onset and PD onset, and (iv) olfactory dysfunction (OSIT-J ≤ 7). The proportions of nociceptive, neuropathic, and nociplastic pain did not differ significantly according to any of these classifications (MIBG: *p* = 0.17; probable RBD: *p* = 0.70; body-first vs brain-first: *p* = 0.29; olfactory dysfunction: *p* = 1.00). These findings suggest that the distribution of PD-related pain subtypes was not strongly associated with these established clinical phenotype markers.

#### 3.2.5 Correlation analysis within each PD-related pain subtype (supplementary Table.3)

Significant positive correlations between PD-PCS scores and MDS-UPDRS Part I item 9 scores (pain and other sensations) were observed in the Nociceptive pain group (*r* = 0.66, *p* < 0.001) and the Neuropathic pain group (*r* = 0.83, *p* < 0.001), indicating that higher PD-PCS scores were associated with greater patient-reported pain burden. PD-PCS scores were also positively correlated with KPPQ total scores in the PD-related pain cohort (*r* = 0.50, *p* < 0.001) and in the Nociceptive pain group (*r* = 0.58, *p* < 0.001), supporting consistency between the PD-PCS and established PD pain questionnaires.

### 3.3 The characteristics of the PD patients evaluated in the second survey (supplementary Table.4)

To comprehensively evaluate the relationship between pain subtypes and motor/non-motor indicators, the second survey newly recruited 40 PD patients with pain who didn't have other identifiable causes of pain. As summarized in Supplementary Table.4, these patients were 22 males (55.0%) and 18 females (45.0%); mean age 65.4 ± 9.2 years, age at onset 54.7 ± 9.6 years, disease duration 10.8 ± 5.9 years, HY stage 2.9 ± 0.9, and LEDD 1005 ± 560 mg. All of these patients underwent evaluations using the full set of motor and non-motor indicators.

### 3.4 Evaluation of PD-related pain using multiple motor and non-motor indicators

#### 3.4.1 The frequency of each pain type (Table.2)

Based on their responses to the PD-PCS questionnaires, the 40 PD patients with pain were categorized into four subgroups: Nociceptive pain (*n* = 24, 60.0%), Neuropathic pain (*n* = 5, 12.5%), Nociplastic pain (*n* = 8, 20.0%), and PD-unrelated pain (*n* = 3, 7.5%) as shown in Table.2.

**Table 2:** Comparison of multiple motor and non-motor indicators among the ‘Nociceptive pain’, ‘Neuropathic pain’, and ‘Nociplastic pain’ group in the second survey.

	PD-related Pain					PD-unrelated Pain
	Nociceptive Pain	Neuropathic Pain	Nociplastic Pain	*p*-value (in PD-related Pain)	Corrected *p*-value (post-hoc)	
Number	24	5	8	–	–	3
Sex (Male: Female)	13: 11	4: 1	3: 5	0.4	–	2: 1
Age	64.3 ± 9.6	65.2 ± 3.8	64.9 ± 10.1	0.98	–	75.7 ± 3.1
Age at onset	54.8 ± 9.8	50.0 ± 6.0	54.1 ± 11.1	0.61	–	63.0 ± 6.2
Disease Duration	9.6 ± 5.5	15.2 ± 4.9	10.8 ± 7.0	0.16	–	12.7 ± 5.7
HY stage	2.7 ± 0.7	2.6 ± 0.5	3.3 ± 1.2	0.47	–	4.3 ± 0.6
LEDD (mg)	878 ± 524	1427 ± 534	1047 ± 515	0.056	–	1200 ± 872
Device aided therapies	DBS 1, LCIG 1	DBS 1, CSCI 1	–	–	–	–
PD-PCS score	36.2 ± 22.5	19.6 ± 16.1	27.5 ± 24.0	0.27	–	NA
KPPS total score	22.5 ± 12.7	20.8 ± 6.2	15.4 ± 9.0	0.32	–	7.0 ± 5.2
1. Musculoskeletal pain	4.8 ± 3.8	3.0 ± 3.7	4.8 ± 4.8	0.69	–	1.7 ± 2.1
2. Chronic pain	3.8 ± 6.3	1.8 ± 2.5	1.3 ± 2.8	0.57	–	2.7 ± 3.1
3. Fluctuation-related pain	5.6 ± 6.0	0.8 ± 1.1	3.1 ± 4.2	0.15	–	0 ± 0
4. Nocturnal pain	4.0 ± 4.8	6.4 ± 5.4	4.5 ± 5.4	0.48	–	1.3 ± 2.3
5. Oro-facial pain	0.8 ± 1.9	1.2 ± 2.7	0.1 ± 0.4	0.73	–	0.7 ± 1.2
6. Discoloration, edema/ swelling	1.2 ± 2.7	2.8 ± 3.4	1.1 ± 2.8	0.39	–	0 ± 0
7. Radicular pain	2.7 ± 3.8	4.8 ± 3.0*	0.5 ± 1.4*	0.029	0.029 (Neuropathic vs Nociplastic)	0.7 ± 0.6
KPPQ total score	4.5 ± 2.2	5.6 ± 2.9	4.1 ± 2.5	0.54	–	3.3 ± 4.2
1. Pain around joints	17 (70.8%)	1 (20.0%)	6 (75.0%)	0.097	–	2 (66.7%)
2. Pain related to a specific internal pain	6 (25.0%)	2 (40.0%)	1 (12.5%)	0.55	–	2 (66.7%)
3. Generalised non-specific pain in stomach area	5 (20.8%)	3 (60.0%)	1 (12.5%)	0.14	–	1 (33.3%)
4. Non-specific pain deep within the body	10 (41.7%)	2 (40.0%)	4 (50.0%)	0.89	–	0
5. Pain related to abnormal involuntary movements	8 (33.3%)	1 (20.0%)	3 (37.5%)	1.00	–	0
6. Painful muscle cramps in a specific region during “off” periods	12 (50.0%)	3 (60.0%)	5 (62.5%)	0.89	–	0
7. Generalised pain during “off” periods	8 (33.3%)	2 (40.0%)	3 (37.5%)	1.00	–	0
8. PLM or RLS-associated pain	11 (45.8%)	3 (60.0%)	1 (12.5%)	0.20	–	1 (33.3%)
9. Pain related to difficulties when turning in bed at night	10 (41.7%)	3 (60.0%)	5 (62.5%)	0.55	–	1 (33.3%)
10. Pain when chewing	2 (8.3%)	1 (20.0%)	0	0.46	–	1 (33.3%)
11. Pain related to grinding teeth during the night	2 (8.3%)	2 (40.0%)	1 (12.5%)	0.18	–	1 (33.3%)
12. Burning sensation in a mouth	2 (8.3%)	1 ((20.0%)	0	0.46	–	0
13. Burning pain in the limbs	3 (12.5%)	1 (20.0%)	1 (12.5%)	0.10	–	0
14. Shooting pain/pins and needles down the limbs	12 (50.0%)	3 (60.0%)	2 (25.0%)	0.49	–	1 (33.3%)
MDS-UPDRS total score	71.7 ± 23.3	70.4 ± 25.1	76.5 ± 32.0	0.88	–	93.0 ± 3.5
MDS-UPDRS Part I total	12.9 ± 8.1	13.6 ± 5.5	16.0 ± 7.3	0.43	–	16.7 ± 4.9
Cognitive impairment	0.5 ± 0.7	1.0 ± 1.4	0.5 ± 0.5	0.82	–	1.7 ± 1.2
Hallucination and psychosis	0.3 ± 0.6	0.8 ± 1.3	1.3 ± 1.4	0.13	–	1.0 ± 1.0
Depressed mood	0.6 ± 0.9	0.8 ± 0.8	0.9 ± 1.1	0.77	–	1.0 ± 1.0
Anxious mood	0.7 ± 0.9	0.4 ± 0.9	0.8 ± 0.7	0.5	–	1.0 ± 1.0
Apathy	0.6 ± 0.9	0.6 ± 1.3	0.1 ± 0.4	0.23	–	1.0 ± 1.0
Features of DDS	0.3 ± 0.6	0.6 ± 0.9	0.3 ± 0.7	0.54	–	0.3 ± 0.6
Sleep problems	1.8 ± 1.3	1.8 ± 0.8	2.0 ± 1.1	0.89	–	1.7 ± 1.2
Daytime sleepiness	1.7 ± 0.9	2.0 ± 1.0	2.4 ± 0.7	0.10	–	2.3 ± 0.6
Pain and other sensations	1.8 ± 1.1	1.4 ± 0.5	1.1 ± 1.0	0.25	–	0.3 ± 0.6
Urinary problems	1.3 ± 1.0	1.2 ± 1.1	1.8 ± 1.0	0.43	–	2.7 ± 1.5
Constipation Problems	1.2 ± 1.0	1.2 ± 1.3	2.1 ± 1.5	0.19	–	2.0 ± 1.0
Light headedness on standing	0.8 ± 1.2	0.6 ± 0.5	1.1 ± 1.0	0.53	–	0.7 ± 0.6
Fatigue	1.3 ± 0.9	1.2 ± 0.8	1.8 ± 1.5	0.71	–	1.0 ± 1.0
MDS-UPDRS Part II	14.9 ± 8.8	16.6 ± 6.1	18.5 ± 8.9	0.58	–	22.3 ± 3.1
MDS-UPDRS Part III	37.8 ± 13.0	30.0 ± 14.3	38.1 ± 17.9	0.53	–	51.7 ± 6.0
MDS-UPDRS Part IV	6.2 ± 4.8	10.2 ± 2.9*	3.9 ± 3.4*	0.032	0.029 (Neuropathic vs Nociplastic)	2.3 ± 3.2
NMSS total	52.6 ± 43.1	60.0 ± 23.4	57.4 ± 30.2	0.50	–	66.0 ± 24.5
Item 27; Pain	7.2 ± 3.3$	3.2 ± 2.7	1.5 ± 2.3$	0.00038	< 0.001 (Nociceptive vs Nociplastic)	0 ± 0
HADS	13.8 ± 9.9	18.6 ± 10.5	12.0 ± 9.1	0.40	–	11.3 ± 5.5
PDSS-2 total	19.8 ± 12.9	18.6 ± 6.1	23.5 ± 14.5	0.84	–	20.0 ± 7.8
Item 4; RLS-related	1.5 ± 1.3	1.2 ± 0.8	1.9 ± 1.6	0.70	–	1.0 ± 0
Item 5; RLS-related	1.0 ± 1.3	1.0 ± 1.0	1.4 ± 1.8	0.94	–	1.3 ± 0.6
Item 10; RLS/nocturnal pain-related	1.0 ± 1.2	0.6 ± 0.9	1.1 ± 1.2	0.75	–	0.7 ± 0.6
Item 11; RLS/nocturnal pain-related	0.9 ± 1.2	0.8 ± 0.4	1.0 ± 1.3	0.89	–	0.7 ± 0.6
Item 12; RLS/nocturnal pain-related	0.9 ± 1.3	1.2 ± 1.6	1.0 ± 1.6	0.81	–	0.7 ± 0.6
PDQ-8 total	9.5 ± 7.1	7.6 ± 2.0	6.1 ± 3.8	0.56	–	11.0 ± 6.9
Item 7; muscle cramps or spasms	1.4 ± 1.3	0.8 ± 0.4	0.4 ± 0.5	0.13	–	0 ± 0
WOQ-9	5.6 ± 1.9	4.6 ± 2.1	6.1 ± 2.2	0.40	–	3.3 ± 3.1
VAS	3554 ± 2548#	540 ± 358#	2206 ± 1545	0.0054	0.0047 (Nociceptive vs Neuropathic)	883 ± 1444

Comparison of multiple motor and non-motor indicators among the ‘Nociceptive pain’, ‘Neuropathic pain’, and ‘Nociplastic pain’ group in the second survey. All data are presented as mean ± standard deviation or n (%). The signs of *, #, and $ means that *p*-value is <0.05, < 0.01, and < 0.001, respectively.

DDS, dopamine dysregulation syndrome; HADS, Hospital Anxiety Depression Rating Scale; HY, Hoehn and Yahr; KPPQ, King's Parkinson's Pain Questionnaire; KPPS, King's Parkinson's Pain Scale; LEDD, levodopa equivalent daily dose; md, mean diffusivity; MDS-UPDRS, Movement Disorder Society Unified Parkinson's Disease Rating Scale; NMSS, Non-Motor Symptoms Scale; PD, Parkinson's disease; PD-PCS, PD Pain Classification System; PDQ-8, Parkinson's Disease Questionnaire-8; PDSS-2, Parkinson's Disease Sleep Scale-2; PLM, periodic limb movement; RLS, restless legs syndrome; VAS, visual analog scale; WOQ-9, 9-item Wearing-off Questionnaire.

#### 3.4.2 Comparisons among the PD-related pain subtypes (Table.2)

Patients in the Neuropathic pain group had higher MDS-UPDRS Part IV scores than those in the Nociplastic pain group (10.2 ± 2.9 vs 3.9 ± 3.4, *p* = 0.029), indicating greater motor complications such as dyskinesia and wearing-off. They also showed more severe radicular pain scores (4.8 ± 3.0 vs 0.5 ± 1.4, *p* = 0.029 vs Nociplastic pain), consistent with a clinically typical neuropathic pain phenotype. Patients in the Nociceptive pain group showed higher NMSS Item 27 (Pain) scores than the Nociplastic pain group (7.2 ± 3.3 vs 1.5 ± 2.3, *p* < 0.001) and higher VAS pain scores (3554 ± 2548 vs 540 ± 358, *p* = 0.0047), suggesting greater subjective pain intensity and symptom burden. No significant differences were found among the three subgroups in other motor or non-motor indicators, including MDS-UPDRS Parts I–III (Part I, *P* = 0.43; Part II, *P* = 0.58; Part III, *p* = 0.53), total NMSS (*p* = 0.50), HADS (*p* = 0.40), PDSS-2 (*p* = 0.84), PDQ-8 (*p* = 0.56), and WOQ-9 (*p* = 0.40). Notably, the Nociplastic pain group showed no significant differences in the RLS-related PDSS-2 Items 4 (*p* = 0.70) and 5 (*p* = 0.94), suggesting that this subtype was not specifically explained by RLS-related symptoms.

#### 3.4.3 Correlation analysis within each PD-related pain subtype (supplementary Table.5)

Significant positive correlations between PD-PCS scores and KPPS total scores were observed in the PD-related pain group (*r* = 0.34, *p* = 0.042) and in the Nociceptive pain group (*r* = 0.43, *p* = 0.035). PD-PCS scores were also positively correlated with VAS pain scores in the PD-related pain group (*r* = 0.55, *p* < 0.001), indicating that higher PD-PCS scores were associated with greater pain severity. In contrast, no significant correlations were observed between PD-PCS and KPPQ total scores in any subgroup. Non-motor symptom burden, represented by MDS-UPDRS Part I and NMSS scores, was significantly correlated with PD-PCS scores in the PD-related pain group (MDS-UPDRS Part I: *r* = 0.42, *p* = 0.0096; NMSS: *r* = 0.38, *p* = 0.021) and in the Nociceptive pain group (MDS-UPDRS Part I: *r* = 0.45, *p* = 0.027; NMSS: *r* = 0.52, *p* = 0.0092), suggesting broader symptom involvement in nociceptive pain. Additional correlations in the Nociceptive pain group were observed for ‘hallucination and psychosis’ (r = 0.45, *p* = 0.028), ‘urinary problems’ (*r* = 0.41, *p* = 0.044) in the MDS-UPDRS Part I, MDS-UPDRS Part II (*r* = 0.46, *p* = 0.024), and total MDS-UPDRS scores (*r* = 0.48, *p* = 0.019), consistent with a wider non-motor and functional burden. In the Neuropathic pain group, PD-PCS scores were strongly correlated with HY stage (*r* = 0.89, *p* = 0.044) and HADS scores (*r* = 0.94, *p* = 0.016), suggesting associations between neuropathic pain severity, disease progression, and affective symptoms.

### 3.5 Comparison between PD patients with and without pain based on MRI findings (supplementary Table.6)

To identify brain regions associated with pain in PD, MRI metrics were compared between patients with pain (*n* = 12) and those without pain (*n* = 15). The upper panel of Supplementary Table. 6 summarizes the clinical characteristics. Patients with pain had higher LEDD (961 ± 612 vs 552 ± 427 mg/day, *p* = 0.026) and higher MDS-UPDRS Part IV scores (4.1 ± 4.8 vs 0.5 ± 1.7, *p* = 0.010), indicating greater treatment complexity and motor complications. Regional morphometric and diffusion analyses identified widespread abnormalities in patients with pain, which remained significant regardless of covariate adjustment (sex, age, and MDS-UPDRS Part IV). These changes primarily involved pain-processing networks, including the caudal/rostral anterior cingulate cortex, inferior/superior parietal lobules, precuneus, supramarginal gyrus, insula, thalamus, and cerebellum. Across these regions, patients with pain generally showed reduced structural integrity (e.g., lower free-water FA or volume and higher diffusivity/free-water-related metrics). Additional abnormalities were observed in regions involved in cognitive-affective pain modulation, including the frontal pole, medial orbitofrontal cortex, fusiform gyrus, cuneus, lingual gyrus, and isthmus of the cingulate gyrus. Overall, these findings suggest more extensive chronic microstructural involvement in distributed sensory, affective, and modulatory pain networks in PD patients with pain.

### 3.6 Comparison among the three PD-related pain types based on MRI findings (Table.3)

To investigate subtype-specific neural correlates, MRI data were compared among patients with nociceptive (*n* = 5), neuropathic (*n* = 3), and nociplastic pain (n = 3). The upper panel of Table. 3 summarizes the clinical characteristics, showing a significant difference only in KPPQ scores (*p* = 0.0041). Regional morphometric and diffusion-based analyses revealed distinct subtype-dependent patterns. The neuropathic pain group demonstrated the most widespread and pronounced microstructural abnormalities, particularly in regions involved in sensory-discriminative processing, including the postcentral gyrus, inferior parietal lobule, supramarginal gyrus, and thalamus proper. Across these regions, alterations were consistent with reduced microstructural integrity, reflected by lower FA-related metrics and higher diffusivity/free-water indices. In addition, abnormalities extended to regions implicated in motor and cognitive-affective pain modulation, including the precentral gyrus, lateral orbitofrontal cortex, isthmus of the cingulate gyrus, and caudate nucleus. These findings suggest that neuropathic pain in PD is associated with multi-network involvement spanning sensory, motor, and modulatory systems. In contrast, the nociplastic pain group showed no consistent or regionally specific abnormalities, supporting the notion that nociplastic pain may not be associated with detectable structural alterations. Notably, the hippocampus exhibited greater impairment in the nociceptive pain group compared with the nociplastic pain group, suggesting a potential role of memory-related processes in nociceptive pain perception. Given the small sample size, these findings should be interpreted as exploratory and hypothesis-generating.

**Table 3:** Comparison of MRI metrics (FreeSurfer, FWI, NODDI) among the ‘Nociceptive pain’, ‘Neuropathic pain’, and ‘Nociplastic pain’ group.

Variables	Nociceptive Pain	Neuropathic Pain	Nociplastic Pain	overall *p*-value (unadj)				
Number	5	3	3	–				
Sex (Male: Female)	2: 3	2: 1	2: 1	1				
Age	71.2 ± 3.6	71 ± 4.6	63 ± 10	0.2				
Age at onset	66 ± 4.7	53.3 ± 11	55.3 ± 4.7	0.062				
Disease Duration	5.2 ± 3.3	17.7 ± 12.3	7.67 ± 5.7	0.11				
HY stage	2.8 ± 0.45	3.33 ± 0.58	2.67 ± 0.58	0.28				
LEDD (mg)	728 ± 265	1230 ± 946	1010 ± 614	0.53				
MDS-UPDRS Part1 Pain and other sensations	2.2 ± 0.84	2.7 ± 0.58	2.0 ± 1.0	0.58				
KPPQ	3.0 ± 1.0#	7.0 ± 1.0#*	3.7 ± 1.5*	0.0041				
PD-PCS score	17.2 ± 17.8	50 ± 19.3	17.7 ± 13.7	0.066				
MMSE	27.6 ± 3.3	22.3 ± 2.3	27.3 ± 1.5	0.12				
MoCA-J	24.6 ± 5.4	20.3 ± 4.9	23.7 ± 2.1	0.48				
MDS-UPDRS Part III	29.2 ± 9.4	47.3 ± 5.7	39.3 ± 23.5	0.25				
MDS-UPDRS Part IV	1.8 ± 2.5	10.7 ± 5.9	4.3 ± 3.8	0.12				

**Brain regions (MRI indicators)**	**Nociceptive Pain**	**Neuropathic Pain**	**Nociplastic Pain**	**overall *p*-value (unadj)**	**overall *p*-value (adj)**	**Neuropathic vs Nociceptive *p*-value (unadj)**	**Neuropathic vs Nociplastic *p*-value (unadj)**	**Nociceptive vs Nociplastic *p*-value (unadj)**
Left hippocampus (freewater ad)	0.0012 ± 0.0001*	0.0011 ± 0.0001	0.0010 ± 0.0001*	0.037	0.015	1.00	0.33	0.031
Left inferior parietal lobule (freewater fa)	0.20 ± 0.01*	0.18 ± 0.01*	0.19 ± 0.01	0.037	0.04	0.031	0.33	1.00
Left isthmus of cingulate gyrus (NODDI ficvf)	0.37 ± 0.01	0.33 ± 0.00*	0.38 ± 0.03*	0.033	0.013	0.088	0.032	0.56
Left lateral orbitofrontal cortex (NODDI ficvf)	0.32 ± 0.0	0.30 ± 0.00*	0.33 ± 0.01*	0.025	0.036	0.06	0.026	0.61
Left postcentral cortex (freewater fa)	0.21 ± 0.01#	0.18 ± 0.00#	0.21 ± 0.01#	0.003	0.0081	0.004	0.0065	1.00
Left precentral gyrus (area)	0.0031 ± 0.0001	0.0028 ± 0.0002*	0.0032 ± 0.0002*	0.021	0.035	0.058	0.02	0.52
Left precentral gyrus								
(freewater fa)	0.24 ± 0.01*	0.21 ± 0.00*	0.23 ± 0.01	0.043	0.0059	0.04	0.26	1.00
Left precentral gyrus (volume)	0.0082 ± 0.0004*	0.0067 ± 0.0005*#	0.0084 ± 0.0007#	0.0055	0.013	0.01	0.0075	0.75
Left superior temporal sulcus and its banks (freewater)	0.32 ± 0.01	0.36 ± 0.03*	0.30 ± 0.04*	0.047	0.034	0.21	0.039	0.33
Left supramarginal gyrus (freewater fa)	0.20 ± 0.01*	0.18 ± 0.00*	0.19 ± 0.01	0.024	0.013	0.019	0.53	0.68
Left thalamus proper (NODDI ficvf)	0.46 ± 0.01*	0.42 ± 0.01*	0.45 ± 0.01	0.037	0.033	0.031	0.33	1.00
Right caudate nucleus (freewater ad)	0.0010 ± 0.0001	0.0012 ± 0.0000*	0.00094 ± 0.0001*	0.024	0.0093	0.21	0.02	0.68
Right caudate nucleus (freewater md)	0.00081 ± 0.00009	0.00096 ± 0.00004*	0.00073 ± 0.00007*	0.015	0.021	0.058	0.013	0.34
Right caudate nucleus (freewater rd)	0.00068 ± 0.00009	0.00083 ± 0.00004*	0.00062 ± 0.00007*	0.02	0.027	0.051	0.02	0.57
Right caudate nucleus (freewater)	0.38 ± 0.03	0.43 ± 0.01*	0.35 ± 0.03*	0.013	0.019	0.07	0.01	0.23
Right inferior parietal lobule (thickness)	2.2 ± 0.0	2.1 ± 0.1#	2.3 ± 0.1#	0.0054	0.0079	0.063	0.0042	0.086
Right lateral orbitofrontal cortex (freewater)	0.32 ± 0.02#	0.37 ± 0.01#	0.31 ± 0.01#	0.0025	0.0058	0.0047	0.0036	0.73
Right transverse temporal gyrus (freewater fa)	0.18 ± 0.01*	0.18 ± 0.02	0.22 ± 0.03*	0.043	0.031	1.00	0.26	0.04

Comparison of MRI metrics (FreeSurfer, FWI, NODDI) among the ‘Nociceptive pain’, ‘Neuropathic pain’, and ‘Nociplastic pain’ group. Sex and age were used as covariates. MRI metrics which showed significant differences regardless of covariates are indicated. All data are presented as mean ± standard deviation or n (%). The signs of * and # means that *p*-value is <0.05, and < 0.01, respectively.

ad, axial diffusivity; fa, fractional anisotropy; ficvf, fitted intracellular volume fraction; fiso, isotropic volume fraction; FWI, free-water imaging; HY, Hoehn and Yahr; KPPQ, King's Parkinson's Pain Questionnaire; LEDD, levodopa equivalent daily dose; md, mean diffusivity; MDS-UPDRS, Movement Disorder Society Unified Parkinson's Disease Rating Scale; MMSE, Mini-Mental State Examination; MoCA-J, Japanese version of the Montreal Cognitive Assessment; MRI, magnetic resonance imaging; NODDI, neurite orientation dispersion and density imaging; PD, Parkinson's disease; PD-PCS, PD Pain Classification System; rd, radial diffusivity.

## 4 Discussion

This study revealed that PD patients with neuropathic pain showed more severe pain intensity (PD-PCS and KPPQ), more severe motor complications (MDS-UPDRS Part IV), and more pronounced MRI abnormalities in brain regions that are directly or indirectly related to pain. In contrast, PD patients with nociplastic pain exhibited no distinct motor or non-motor characteristics, no evident MRI abnormalities, and no correlations with their PD-PCS scores. The proportions of each pain type among the patients with pain (*n* = 107 in the first survey; *n* = 40 in the second) were as follows: nociceptive pain (37.4%, 60.0%), neuropathic pain (18.7%, 12.5%), nociplastic pain (23.4%, 20.0%), and PD-unrelated pain (20.6%, 7.5%). Aside from the exclusion of patients with other identifiable pain causes from the second survey (which reduced the proportion of patients with PD-unrelated pain), these results are comparable to those of the PD-PCS validation study: nociceptive (55%), neuropathic (16%), nociplastic (22%), and PD-unrelated pain (23%) [4]. This consistency in the frequency with the previous study suggested the validity of this research, confirming that the nociplastic pain is observed in a significant number of PD patients.

A review described nociplastic pain in PD as an abnormal gain in nociceptive processing without somatosensory or tissue pathology [23]. Unlike neuropathic or nociceptive pain, nociplastic pain in PD is often poorly localized, periabdominal, diffuse, or variable in location, and it appears as part of broader, more complex PD features [24]. In the web-based PD-PCS, nociplastic pain is determined after excluding nociceptive and neuropathic pain, whereas the validation study evaluated each PD-related pain type separately. Because nociplastic pain in PD is thought to arise from hyper−/hypodopaminergic fluctuations, neuropsychiatric manifestations, and/or central sensitization, factors such as the non-motor off state, RLS, DAWS, and DDS are originally incorporated into the nociplastic pain section of the PD-PCS [4]. However, these findings didn't indicate that these motor or non-motor fluctuations can distinguish PD-related nociplastic pain from the other pain type. More detailed assessments such as the Movement Disorder Society Non-Motor Rating Scale or the International Restless Legs Syndrome Study Group Severity Rating Scale are desired.

In direct pain processing, the postcentral gyrus (primary somatosensory cortex) plays a central role in pain perception and localization [25], and the thalamus serves as a major nociceptive relay, integrating pain signals before cortical projection [26]. The inferior/superior parietal lobules and precuneus/supramarginal gyrus contribute to sensory-discriminative and integrative pain functions [27]. The orbitofrontal cortex modulates pain through executive and affective control, and the isthmus of the cingulate gyrus regulates memory- and emotion-related modulation [26]. In this MRI analysis, brain regions that are implicated in pain causation or modulation were more affected in the PD patients with pain, especially those with neuropathic pain. More pronounced abnormalities were observed in the caudate nucleus in the Neuropathic pain group, whereas the hippocampus was more affected in the Nociceptive pain group. Considering prior reports showing the caudate nucleus's involvement in chronic and neuropathic pain and the interaction between nociceptive input and hippocampal function in chronic musculoskeletal pain, these regions may influence the pain phenotype [28], [29]. Considering that the nociplastic pain consistently lacked distinct structural changes in the present MRI data, this pain is highly likely to be functional in nature, potentially requiring functional MRI analysis to elucidate its characteristics.

This study has several limitations. First, the web-based PD-PCS used in this study assigns a single dominant pain type and does not allow classification of overlapping pain types. However, previous studies have reported that PD-related pain frequently overlaps; Mylius et al. reported overlapping pain types in 14% of patients (predominantly neuropathic–nociceptive overlap) and all three types in 1% [4]. Given that pain rarely occurs in isolation in clinical practice, the lack of overlap classification in the present study may have influenced the observed distribution and characterization of pain subtypes. Future studies incorporating overlapping pain categories will be important to better reflect the complexity of PD-related pain. Second, the PD-PCS content was translated into Japanese by an author involved in the translation of the KPPS and KPPQ [20], but not according to standardized linguistic validation procedures [30] or the MDS Translation Program. To mitigate this limitation, the instrument was administered with physicians' supplementary explanations. Notably, after completion of this study, a formally validated Japanese version of the PD-PCS was published by another institution in Japan [31], which will facilitate more standardized assessments in future studies. Third, the number of patients in each pain subgroup was small, particularly in the neuropathic pain group of the second survey and MRI subgroup. Therefore, these findings should be interpreted with caution and require validation in larger cohorts. To provide complementary information, multiple MRI metrics (FreeSurfer, FWI, and NODDI) were incorporated; however, these imaging findings remain supplementary and exploratory. Finally, the timing of PD-PCS evaluation and MRI acquisition was not identical (within 1 year). However, more than half of the MRI scans were obtained within 6 months of the clinical assessments, and no marked HY stage progression was observed during the interval. Moreover, the PD-PCS validation study showed a stable pain type classification across patient visits without relevant clinical changes [4], suggesting that this time lag is acceptable.

In conclusion, PD-related pain was characterized using the PD-PCS, supported by supplementary MRI analysis. These analysis results demonstrated that neuropathic pain in PD represents a severe phenotype linked to motor fluctuations and structural brain changes, requiring more focused intervention. These findings suggest that targeted treatment strategies, including medications for neuropathic pain and optimization of dopaminergic therapy, may be beneficial. Furthermore, this study provides the first PD-PCS-based characterization of nociplastic pain, highlighting its diagnostic complexity. The lack of traditional clinical features and MRI structural changes in the nociplastic pain suggests that current assessment tools may under-capture this specific pain mechanism, underscoring the need for specialized multidimensional diagnostic approaches in combination with the PC-PCS.

## Abbreviations

**Table:** 

ad	axial diffusivity
ANCOVA	analysis of covariance
DAWS	dopamine agonist withdrawal syndrome
DDS	dopamine dysregulation syndrome
DWI	diffusion-weighted imaging
EPI	echo-planer imaging
fa	fractional anisotropy
FA	flip angle
ficvf	fitted intracellular volume fraction
fiso	isotropic volume fraction
FWI	free-water imaging
HADS	Hospital Anxiety Depression Rating Scale
HY	Hoehn and Yahr
KPPQ	King's Parkinson's Pain Questionnaire
KPPS	King's Parkinson's Pain Scale
LEDD	levodopa equivalent daily dose
md	mean diffusivity
MDS	Movement Disorder Society
MDS-UPDRS	Movement Disorder Society Unified Parkinson's Disease Rating Scale
MIBG	metaiodobenzylguanidine
MMSE	Mini-Mental State Examination
MoCA-J	Japanese version of the Montreal Cognitive Assessment
MRI	magnetic resonance imaging
NMSS	Non-Motor Symptoms Scale
NODDI	neurite orientation dispersion and density imaging
OSIT-J	Odor Stick Identification Test for Japanese
PD	Parkinson's disease
PD-PCS	PD Pain Classification System
PDQ-8	Parkinson's Disease Questionnaire-8
PDSS-2	Parkinson's Disease Sleep Scale-2
RBD	REM sleep behavior disorder
RBDSQ-J	Japanese version of the REM Sleep Behavior Disorder Screening Questionnaire
rd	radial diffusivity
RLS	restless legs syndrome
TE	echo time
TR	repetition time
VAS	visual analog scale
WOQ-9	9-item Wearing-off Questionnaire.

## Ethical compliance statement

This study design and protocol were approved by the Ethics Committee for Human Research of the Keio University School of Medicine (#N20231166). Written informed consent was waived in accordance with national ethical guidelines, and an opt-out procedure was implemented by disclosing study information on the institutional website to allow participants the opportunity to decline inclusion. We confirm that we have read the Journal's position on issues involved in ethical publication and affirm that this work is consistent with those guidelines.

## CRediT authorship contribution statement

**Toshiki Tezuka:** Writing – review & editing, Writing – original draft, Validation, Project administration, Methodology, Investigation, Formal analysis, Data curation, Conceptualization. **Tomonori Nukariya:** Writing – review & editing, Validation, Investigation, Data curation. **Shohei Okusa:** Writing – review & editing, Validation, Investigation. **Ryo Ueda:** Writing – review & editing, Validation, Methodology, Investigation, Formal analysis, Data curation. **Hiroki Saegusa:** Writing – review & editing, Validation, Investigation. **Ryotaro Okochi:** Writing – review & editing, Validation, Investigation. **Yuto Sakai:** Writing – review & editing, Validation. **Yoshihiro Nihei:** Writing – review & editing, Supervision. **Jin Nakahara:** Writing – review & editing, Supervision. **Morinobu Seki:** Writing – review & editing, Supervision, Resources, Project administration, Methodology, Funding acquisition, Data curation, Conceptualization.

## Funding source

This work was supported in part by the Japan Agency for Medical Research and Development (AMED) under grant numbers JP22K07501 and JP25K10775 (MS). The other authors declare no conflicts of interest relevant to this work.

## Declaration of competing interest

The authors declare that they have no known competing financial interests or personal relationships that could have appeared to influence the work reported in this paper.
