# Supplementary material for: The characterization of Parkinson's disease related pain based on the classification system: The severity of neuropathic pain and the clinical profile of nociplastic pain
Source: Clin Park Relat Disord. 2026 Jul 27;15:100490. doi: 10.1016/j.prdoa.2026.100490 (PMC13463971; doi:10.1016/j.prdoa.2026.100490)
Supplement: Supplementary file 1 [file mmc1.docx]

Supplementary Table.1

| Characteristics | Values |  |  |
| --- | --- | --- | --- |
| Number | N = 192 |  |  |
| Sex (Male/Female) | 112 (58.3%)/80 (41.7%) |  |  |
| Age | 72.7 ± 8.9 |  |  |
| Age at onset | 64.5 ± 10.1 |  |  |
| Disease duration | 8.2 ± 5.8 |  |  |
| HY Stage | 2.8 ± 0.9 |  |  |
| HY Stage 1/2/3/4/5 | 14 (7.3%)/54 (28.1%)/80 (41.7%)/38 (19.8%)/6 (3.1%) |  |  |
| LEDD (mg) | 690 ± 439 |  |  |
| Rating for each scale | |  |  |
| Variables | | n | Values |
| KPPQ total score | | 192 | 2.0 ± 2.5 |
| 1. Pain around joints | |  | 86 (44.8%) |
| 2. Pain related to a specific internal pain | |  | 23 (12.0%) |
| 3. Generalised non-specific pain in stomach area | |  | 20 (10.4%) |
| 4. Non-specific pain deep within the body | |  | 25 (13.0%) |
| 5. Pain related to abnormal involuntary movements | |  | 17 (8.9%) |
| 6. Painful muscle cramps in a specific region during “off” periods | |  | 41 (21.4%) |
| 7. Generalised pain during “off” periods | |  | 22 (11.5%) |
| 8. PLM or RLS-associated pain | |  | 45 (23.4%) |
| 9. Pain related to difficulties when turning in bed at night | |  | 33 (17.2%) |
| 10. Pain when chewing | |  | 9 (4.7%) |
| 11. Pain related to grinding teeth during the night | |  | 4 (2.1%) |
| 12. Burning sensation in a mouth | |  | 6 (3.1%) |
| 13. Burning pain in the limbs | |  | 13 (6.8%) |
| 14. Shooting pain/pins and needles down the limbs | |  | 35 (18.2%) |
| MDS-UPDRS Part Ⅰ; Pain and other sensations | |  | 1.1 ± 1.2 |
| MMSE | | 79 | 27.0 ± 3.2 |
| MoCA-J | | 79 | 23.1 ± 4.0 |
| OSIT-J | | 151 | 4.0 ± 2.9 |
| RBDSQ-J | | 172 | 4.8 ± 3.1 |
| MIBG scintigraphy | | 162 | pos: 133, neg: 29 |

Demographic data of PD patients evaluated in the first survey. All data are presented as mean ± standard deviation or n (%).

HY, Hoehn and Yahr; KPPQ, King’s Parkinson’s Pain Questionnaire; LEDD, levodopa equivalent daily dose; MDS-UPDRS, Movement Disorder Society Unified Parkinson’s Disease Rating Scale; MIBG, metaiodobenzylguanidine; MMSE, Mini-Mental State Examination; MoCA-J, Japanese version of the Montreal Cognitive Assessment; OSIT-J, Odor Stick Identification Test for Japanese; PD, Parkinson’s disease; PLM, periodic limb movement; RBDSQ-J, Japanese version of the REM Sleep Behavior Disorder Screening Questionnaire; RLS, restless legs syndrome.

Supplementary Table.2

|  | | PD-related Pain | | | |
| --- | --- | --- | --- | --- | --- |
|  |  | Nociceptive Pain | Neuropathic Pain | Nociplastic Pain | *p*-value (in PD-related Pain) |
| MIBG scintigraphy | + | 31 (49.2%) | 16 (25.4%) | 16 (25.4%) | 0.17 |
|  | - | 4 (36.4%) | 1 (9.1%) | 6 (54.5%) |  |
| Probable RBD | + | 15 (45.5%) | 9 (27.3%) | 9 (27.3%) | 0.70 |
|  | - | 18 (42.9%) | 9 (21.4%) | 15 (35.7%) |  |
| Body-first / Brain-first | Body-first | 11 (57.9%) | 4 (21.1%) | 4 (21.1%) | 0.29 |
|  | Brain-first | 4 (28.6%) | 5 (35.7%) | 5 (35.7%) |  |
| Olfactory dysfunction | + | 27 (48.2%) | 13 (23.2%) | 16 (28.6%) | 1.00 |
|  | - | 4 (57.1%) | 1 (14.3%) | 2 (28.6%) |  |

Comparison of the proportions of the ‘Nociceptive pain’, ‘Neuropathic pain’, and ‘Nociplastic pain’ based on each categorization. MIBG scintigraphy was classified as positive when the heart-to-mediastinum uptake ratio was < 2.2, and negative when the ratio was ≥ 2.2. Probable RBD was defined as 6 or more of RBDSQ-J in the first survey. Body-first PD was defined as PD patients with RBD who developed RBD before the onset of PD, whereas brain-first PD was defined as PD patients with RBD who developed RBD after the onset of PD. Olfactory dysfunction was defined as 7 or less of OSIT-J.

MIBG, metaiodobenzylguanidine; OSIT-J, Odor Stick Identification Test for Japanese; PD, Parkinson’s disease; RBD, REM sleep behavior disorder; RBDSQ-J, Japanese version of the REM Sleep Behavior Disorder Screening Questionnaire.

Supplementary Table.3

| Variables | Correlation with PD-PCS score | | | | | | | |
| --- | --- | --- | --- | --- | --- | --- | --- | --- |
|  | Nociceptive Pain | | Neuropathic Pain | | Nociplastic Pain | | PD related Pain | |
|  | r | *p*-value | r | *p*-value | r | *p*-value | r | *p*-value |
| Age | 0.082 | 0.62 | -0.11 | 0.65 | -0.13 | 0.53 | -0.009 | 0.94 |
| Age at onset | 0.086 | 0.6 | -0.37 | 0.11 | -0.052 | 0.81 | -0.044 | 0.69 |
| Disease duration | -0.0013 | 0.99 | 0.26 | 0.27 | -0.13 | 0.54 | 0.029 | 0.79 |
| HY stage | 0.26 | 0.10 | 0.18 | 0.45 | 0.027 | 0.90 | 0.21 | 0.059 |
| LEDD (mg) | -0.044 | 0.79 | 0.20 | 0.39 | -0.21 | 0.30 | 0.013 | 0.91 |
| MDS-UPDRS Part Ⅰ; Pain and other sensations | 0.66$ | < 0.001 | 0.83$ | < 0.001 | 0.39 | 0.053 | 0.62$ | < 0.001 |
| KPPQ total score | 0.58$ | < 0.001 | 0.40 | 0.079 | -0.075 | 0.72 | 0.50$ | < 0.001 |
| MMSE | -0.20 | 0.46 | -0.36 | 0.34 | -0.11 | 0.74 | -0.042 | 0.81 |
| MoCA-J | -0.054 | 0.84 | -0.051 | 0.90 | 0.44 | 0.15 | 0.19 | 0.26 |
| OSIT-J | 0.12 | 0.52 | -0.11 | 0.71 | 0.38 | 0.12 | 0.18 | 0.15 |
| RBDSQ-J | 0.095 | 0.60 | 0.06 | 0.81 | -0.074 | 0.73 | 0.046 | 0.70 |

Correlation coefficients with PD-PCS scores within each PD-related pain subtype in the first survey. The sign of $ means that *p*-value is < 0.001.

HY, Hoehn and Yahr; KPPQ, King’s Parkinson’s Pain Questionnaire; LEDD, levodopa equivalent daily dose; MDS-UPDRS, Movement Disorder Society Unified Parkinson’s Disease Rating Scale; MMSE, Mini-Mental State Examination; MoCA-J, Japanese version of the Montreal Cognitive Assessment; OSIT-J, Odor Stick Identification Test for Japanese; PD, Parkinson’s disease; PD-PCS, PD Pain Classification System; RBDSQ-J, Japanese version of the REM Sleep Behavior Disorder Screening Questionnaire.

.

Supplementary Table.4

| Characteristics | Values |
| --- | --- |
| Number | N = 40 |
| Sex (Male/Female) | 22 (55%)/18 (45%) |
| Age | 65.4 ± 9.2 |
| Age at onset | 54.7 ± 9.6 |
| Disease duration | 10.8 ± 5.9 |
| HY Stage | 2.9 ± 0.9 |
| HY Stage 1/2/3/4/5 | 0 (0%)/14 (35%)/18 (45%)/5 (12.5%)/3 (7.5%) |
| LEDD (mg) | 1005 ± 560 |
| Device aided therapies | CSCI 1, DBS 2, LCIG 1 |
| Rating for each scale |  |
| Variables | Values |
| KPPS total score | 19.7 ± 11.7 |
| KPPQ total score | 4.5 ± 2.5 |
| MDS-UPDRS total score | 74.1 ± 24.6 |
| MDS-UPDRS Part Ⅲ | 37.9 ± 14.2 |
| NMSS | 55.5 ± 36.9 |
| HADS | 13.9 ± 9.4 |
| PDSS-2 | 20.4 ± 12.1 |
| PDQ-8 | 8.7 ± 6.1 |
| WOQ-9 | 5.4 ± 2.1 |
| VAS | 2707 ± 2393 |

Demographic data of PD patients with pain evaluated by using multiple motor and non-motor indicators in the second survey. All data are presented as mean ±standard deviation or n (%).

CSCI, continuous subcutaneous infusion; DBS, deep brain stimulation; HY, Hoehn and Yahr; KPPQ, King’s Parkinson’s Pain Questionnaire; KPPS, King’s Parkinson’s Pain Scale; LCIG, levodopa-carbidopa intestinal gel; LEDD, levodopa equivalent daily dose; MDS-UPDRS, Movement Disorder Society Unified Parkinson’s Disease Rating Scale; NMSS, Non-Motor Symptoms Scale; PD, Parkinson’s disease; PDQ-8, Parkinson’s Disease Questionnaire-8; PDSS-2, Parkinson’s Disease Sleep Scale-2; VAS, visual analog scale; WOQ-9, 9-item Wearing-off Questionnaire.

Supplementary Table.5

| Variables | Correlation with PD-PCS score | | | | | | | |
| --- | --- | --- | --- | --- | --- | --- | --- | --- |
|  | Nociceptive Pain | | Neuropathic Pain | | Nociplastic Pain | | PD related Pain | |
|  | r | *p*-value | r | *p*-value | r | *p*-value | r | *p*-value |
| Age | 0.34 | 0.10 | 0.72 | 0.18 | -0.43 | 0.29 | 0.15 | 0.39 |
| Age at onset | 0.33 | 0.12 | 0.11 | 0.86 | -0.34 | 0.41 | 0.18 | 0.29 |
| Disease Duration | 0.015 | 0.95 | 0.43 | 0.48 | -0.084 | 0.84 | -0.066 | 0.70 |
| LEDD (mg) | 0.09 | 0.68 | 0.38 | 0.53 | 0.43 | 0.29 | 0.056 | 0.74 |
| HY stage | 0.25 | 0.25 | 0.89* | 0.044 | -0.51 | 0.20 | 0.10 | 0.57 |
| KPPS total score | 0.43* | 0.035 | -0.38 | 0.53 | 0.52 | 0.19 | 0.34* | 0.042 |
| 1. Musculoskeletal pain | 0.18 | 0.41 | -0.42 | 0.48 | -0.15 | 0.72 | 0.063 | 0.71 |
| 2. Chronic pain | 0.36 | 0.083 | 0.70 | 0.19 | 0.32 | 0.44 | 0.40* | 0.014 |
| 3. Fluctuation-related pain | 0.36 | 0.083 | -0.59 | 0.29 | -0.012 | 0.98 | 0.28 | 0.095 |
| 4. Nocturnal pain | -0.034 | 0.88 | -0.84 | 0.073 | 0.039 | 0.93 | -0.11 | 0.52 |
| 5. Oro-facial pain | -0.0026 | 0.99 | 0.54 | 0.34 | 0.17 | 0.69 | 0.11 | 0.51 |
| 6. Discoloration, edema/swelling | -0.085 | 0.70 | 0.55 | 0.33 | 0.15 | 0.72 | 0.069 | 0.69 |
| 7. Radicular pain | 0.026 | 0.90 | -0.26 | 0.68 | -0.42 | 0.30 | -0.045 | 0.79 |
| MDS-UPDRS Part Ⅰtotal | 0.45* | 0.027 | 0.90* | 0.039 | 0.17 | 0.69 | 0.42# | 0.0096 |
| Cognitive impairment | 0.28 | 0.18 | 0.86 | 0.061 | 0.28 | 0.51 | 0.32 | 0.052 |
| Hallucination and psychosis | 0.45* | 0.028 | 0.86 | 0.061 | -0.0043 | 0.99 | 0.29 | 0.082 |
| Depressed mood | 0.25 | 0.24 | 0.59 | 0.30 | 0.42 | 0.31 | 0.25 | 0.13 |
| Anxious mood | 0.26 | 0.23 | 0.54 | 0.34 | 0.10 | 0.81 | 0.30 | 0.074 |
| Apathy | 0.072 | 0.74 | 0.54 | 0.34 | 0.17 | 0.69 | 0.20 | 0.23 |
| Features of DDS | 0.048 | 0.82 | 0.86 | 0.061 | -0.42 | 0.30 | 0.067 | 0.70 |
| Sleep problems | 0.25 | 0.25 | -0.38 | 0.53 | -0.067 | 0.88 | 0.09 | 0.60 |
| Daytime sleepiness | 0.15 | 0.47 | -0.22 | 0.73 | 0.23 | 0.58 | 0.059 | 0.73 |
| Pain and other sensations | 0.28 | 0.18 | 0.44 | 0.45 | -0.18 | 0.67 | 0.26 | 0.12 |
| Urinary problems | 0.41* | 0.044 | -0.73 | 0.16 | 0.098 | 0.82 | 0.14 | 0.41 |
| Constipation Problems | 0.36 | 0.087 | 0.34 | 0.58 | 0.10 | 0.81 | 0.24 | 0.16 |
| Light headedness on standing | 0.015 | 0.95 | 0.30 | 0.63 | 0.64 | 0.089 | 0.17 | 0.32 |
| Fatigue | 0.53 | 0.0074 | 0.6 | 0.28 | 0.28 | 0.51 | 0.45# | 0.0056 |
| MDS-UPDRS Part Ⅱ | 0.46* | 0.024 | 0.32 | 0.61 | -0.41 | 0.32 | 0.20 | 0.24 |
| MDS-UPDRS Part Ⅲ | 0.19 | 0.37 | 0.72 | 0.17 | -0.28 | 0.50 | 0.14 | 0.42 |
| MDS-UPDRS Part Ⅳ | 0.15 | 0.48 | 0.51 | 0.38 | 0.032 | 0.94 | 0.00024 | 1.00 |
| MDS-UPDRS total score | 0.48* | 0.019 | 0.74 | 0.15 | -0.22 | 0.61 | 0.28 | 0.091 |
| NMSS total | 0.52# | 0.0092 | 0.42 | 0.48 | 0.45 | 0.27 | 0.38* | 0.021 |
| Item 27; Pain | 0.058 | 0.79 | 0.13 | 0.84 | 0.026 | 0.95 | 0.28 | 0.096 |
| WOQ-9 | 0.32 | 0.13 | 0.83 | 0.08 | -0.025 | 0.95 | 0.27 | 0.10 |
| PDQ-8 total | 0.33 | 0.12 | -0.057 | 0.93 | -0.28 | 0.50 | 0.20 | 0.23 |
| Item 7; muscle cramps or spasms | 0.30 | 0.15 | 0.36 | 0.55 | 0.17 | 0.68 | 0.33 | 0.05 |
| VAS | 0.40 | 0.056 | 0.86 | 0.059 | 0.56 | 0.15 | 0.55$ | < 0.001 |
| PDSS-2 total | 0.24 | 0.26 | -0.37 | 0.54 | 0.20 | 0.63 | 0.11 | 0.52 |
| Item 4; RLS-related | 0.34 | 0.10 | -0.33 | 0.59 | 0.29 | 0.49 | 0.27 | 0.11 |
| Item 5; RLS-related | 0.057 | 0.79 | -0.25 | 0.69 | 0.36 | 0.38 | 0.057 | 0.74 |
| Item 10; RLS/nocturnal pain-related | 0.28 | 0.18 | -0.34 | 0.57 | 0.22 | 0.60 | 0.20 | 0.24 |
| Item 11; RLS/nocturnal pain-related | 0.36 | 0.082 | -0.54 | 0.34 | 0.25 | 0.55 | 0.24 | 0.16 |
| Item 12; RLS/nocturnal pain-related | 0.21 | 0.33 | -0.64 | 0.25 | 0.39 | 0.34 | 0.11 | 0.51 |
| HADS | -0.0042 | 0.99 | 0.94* | 0.016 | 0.38 | 0.35 | 0.21 | 0.22 |
| KPPQ total score | 0.23 | 0.27 | 0.44 | 0.46 | 0.50 | 0.21 | 0.27 | 0.11 |

Correlation coefficients with PD-PCS scores within each PD-related pain subtype in the second survey. The signs of *, #, and $ means that *p*-value is < 0.05, < 0.01, and < 0.001, respectively.

DDS, dopamine dysregulation syndrome; HADS, Hospital Anxiety Depression Rating Scale; HY, Hoehn and Yahr; KPPQ, King’s Parkinson’s Pain Questionnaire; KPPS, King’s Parkinson’s Pain Scale; LEDD, levodopa equivalent daily dose; MDS-UPDRS, Movement Disorder Society Unified Parkinson’s Disease Rating Scale; NMSS, Non-Motor Symptoms Scale; PD, Parkinson’s disease; PD-PCS, PD Pain Classification System; PDQ-8, Parkinson’s Disease Questionnaire-8; PDSS-2, Parkinson’s Disease Sleep Scale-2; RLS, restless legs syndrome; VAS, visual analog scale; WOQ-9, 9-item Wearing-off Questionnaire.

.

Supplementary Table.6

| **Variables** | **PD without pain** | **PD with pain** | ***p*-value** |  |
| --- | --- | --- | --- | --- |
| Number | 12 | 15 | - |  |
| Sex (Male : Female) | 9 : 3 | 10 : 5 | 0.70 |  |
| Age | 71.2 ± 4.2 | 70.3 ± 6.9 | 0.67 |  |
| Age at onset | 65.2 ± 5.0 | 61.4 ± 9.3 | 0.18 |  |
| Disease Duration | 6 ± 4.5 | 8.9 ± 8.0 | 0.42 |  |
| HY stage | 2.7 ± 0.65 | 3 ± 0.85 | 0.32 |  |
| LEDD (mg) | 552 ± 427* | 961 ± 612* | 0.026 |  |
| MDS-UPDRS Part1 Pain and other sensations | 0 | 1.9 ± 1.1 | - |  |
| KPPQ | 0 | 3.5 ± 2.3 | - |  |
| PD-PCS score | 0 | 22 ± 21 | - |  |
| MMSE | 27.1 ± 3.0 | 26.5 ± 3.3 | 0.69 |  |
| MoCA-J | 23.6 ± 3.8 | 24 ± 4.2 | 0.79 |  |
| MDS-UPDRS Part Ⅲ | 30.4 ± 15.9 | 39.1 ± 14.3 | 0.16 |  |
| MDS-UPDRS Part Ⅳ | 0.5 ± 1.7 | 4.1 ± 4.8 | 0.01 |  |
| **Brain regions (MRI indicators)** | **PD without pain** | **PD with pain** | ***p*-value (unadj)** | ***p*-value (adj)** |
| Left caudal anterior cingulate cortex (freewater fa) | 0.27 ± 0.03* | 0.24 ± 0.02* | 0.016 | 0.022 |
| Left cerebellum cortex (freewater fa) | 0.26 ± 0.02* | 0.23 ± 0.03* | 0.043 | 0.018 |
| Left cuneus gyrus (freewater fa) | 0.17 ± 0.02* | 0.15 ± 0.02* | 0.019 | 0.027 |
| Left frontal pole (freewater fa) | 0.22 ± 0.04* | 0.19 ± 0.03* | 0.029 | 0.011 |
| Left frontal pole (NODDI ficvf) | 0.3 ± 0.05* | 0.26 ± 0.04* | 0.026 | 0.018 |
| Left fusiform gyrus (freewater fa) | 0.22 ± 0.03* | 0.19 ± 0.02* | 0.032 | 0.041 |
| Left inferior parietal lobule (freewater fa) | 0.20 ± 0.02* | 0.19 ± 0.01* | 0.038 | 0.025 |
| Left insula lobe (freewater fa) | 0.25 ± 0.02* | 0.24 ± 0.01 | 0.038 | 0.023 |
| Left insula lobe (freewater rd) | 0.00063 ± 0.00003* | 0.00068 ± 0.00005* | 0.006 | 0.030 |
| Left isthmus of cingulate gyrus (freewater rd) | 0.00065 ± 0.00003* | 0.00068 ± 0.00006* | 0.048 | 0.031 |
| Left lateral occipital sulcus (freewater fa) | 0.19 ± 0.03* | 0.17 ± 0.01* | 0.011 | 0.0036 |
| Left lingual gyrus (freewater fa) | 0.18 ± 0.03* | 0.16 ± 0.01* | 0.031 | 0.026 |
| Left medial orbitofrontal cortex (freewater fa) | 0.25 ± 0.03# | 0.22 ± 0.04# | 0.0051 | 0.0084 |
| Left medial orbitofrontal cortex (freewater rd) | 0.00065 ± 0.00004* | 0.00071 ± 0.00007* | 0.022 | 0.042 |
| Left parahippocampal cortex (freewater fa) | 0.24 ± 0.04* | 0.21 ± 0.03* | 0.028 | 0.027 |
| Left pars opercularis (freewater rd) | 0.00071 ± 0.00004* | 0.00075 ± 0.00004* | 0.019 | 0.047 |
| Left precuneus gyrus (freewater fa) | 0.20 ± 0.02# | 0.18 ± 0.01# | 0.0016 | < 0.001 |
| Left superior parietal lobule (freewater fa) | 0.21 ± 0.02* | 0.19 ± 0.01* | 0.022 | 0.019 |
| Left supramarginal gyrus (freewater fa) | 0.21 ± 0.02* | 0.19 ± 0.01* | 0.0060 | 0.023 |
| Right cuneus gyrus (freewater fa) | 0.17 ± 0.021# | 0.15 ± 0.014# | 0.0031 | 0.002 |
| Right inferior parietal lobule (freewater fa) | 0.20 ± 0.02* | 0.19 ± 0.01* | 0.045 | 0.0053 |
| Right medial orbitofrontal cortex (NODDI fiso) | 0.15 ± 0.03* | 0.19 ± 0.05* | 0.018 | 0.034 |
| Right pars triangularis (freewater fa) | 0.20 ± 0.03* | 0.18 ± 0.01* | 0.013 | 0.0093 |
| Right rostral anterior cingulate cortex (volume) | 0.0012 ± 0.0002* | 0.0011 ± 0.0002* | 0.041 | 0.016 |
| Right thalamus proper (freewater ad) | 0.00097 ± 0.00005* | 0.0010 ± 0.00009* | 0.025 | 0.021 |
| Right thalamus proper (NODDI fiso) | 0.20 ± 0.03* | 0.25 ± 0.06* | 0.026 | 0.04 |
| Right thalamus proper (freewater md) | 0.00065 ± 0.00004* | 0.00071 ± 0.00007* | 0.013 | 0.017 |
| Right thalamus proper (freewater rd) | 0.00049 ± 0.00004* | 0.00055 ± 0.00007* | 0.01 | 0.021 |
| Right whole thalamus (volume) | 0.0040 ± 0.0002* | 0.0038 ± 0.0003* | 0.0060 | 0.049 |
| Optic chiasm (volume) | 0.00016 ± 0.00001* | 0.00013 ± 0.00003* | 0.023 | 0.029 |

Comparison of MRI metrics (FreeSurfer, FWI, NODDI) in PD patients with or without pain. Sex, age and MDS-UPDRS Part Ⅳ were used as covariates. MRI metrics which showed significant differences regardless of covariates are indicated. All data are presented as mean ±standard deviation or n (%). The signs of * and # means that *p*-value is < 0.05 and < 0.01, respectively.

ad, axial diffusivity; fa, fractional anisotropy; FA, flip angle; ficvf, fitted intracellular volume fraction; fiso, isotropic volume fraction; FWI, free-water imaging; HY, Hoehn and Yahr; KPPQ, King’s Parkinson’s Pain Questionnaire; LEDD, levodopa equivalent daily dose; md, mean diffusivity; MDS-UPDRS, Movement Disorder Society Unified Parkinson’s Disease Rating Scale; MMSE, Mini-Mental State Examination; MoCA-J, Japanese version of the Montreal Cognitive Assessment; MRI, magnetic resonance imaging; NODDI, neurite orientation dispersion and density imaging; PD, Parkinson’s disease; PD-PCS, PD Pain Classification System; rd, radial diffusivity.
